# miR-23b-3p, miR-124-3p and miR-218-5p Synergistic or Additive Effects on Cellular Processes That Modulate Cervical Cancer Progression? A Molecular Balance That Needs Attention

**DOI:** 10.3390/ijms232113551

**Published:** 2022-11-04

**Authors:** Manuel Joaquín Romero-López, Hilda Jiménez-Wences, Merlin Itsel Cruz-De la Rosa, Ilce Valeria Román-Fernández, Gloria Fernández-Tilapa

**Affiliations:** 1 Laboratorio de Investigación Clínica, Facultad de Ciencias Químico Biológicas, Universidad Autónoma de Guerrero, Avenida Lázaro Cárdenas S/N, Ciudad Universitaria Sur, Col. La Haciendita, Chilpancingo 39087, Guerrero, Mexico; 2 Laboratorio de Investigación en Biomoléculas, Facultad de Ciencias Químico Biológicas, Universidad Autónoma de Guerrero, Avenida Lázaro Cárdenas S/N, Ciudad Universitaria Sur, Col. La Haciendita, Chilpancingo 39087, Guerrero, Mexico; 3 Instituto de Investigación en Ciencias Biomédicas, Centro Universitario de Ciencias de la Salud, Universidad de Guadalajara, Guadalajara 44340, Jalisco, Mexico

**Keywords:** miRNAs, cervical cancer, bioinformatic analysis, miR-23b-3p, miR-124-3p, miR-218-5p

## Abstract

In cervical cancer (CC), miR-23b-3p, miR-124-3p, and miR-218-5p have been found to act as tumor suppressors by regulating cellular processes related to progression and metastasis. The objective of the present review is to provide an update on the experimental evidence about the role of miR-23b-3p, miR-124-3p, and miR-218-5p in the regulation of CC progression. Additionally, we present the results of a bioinformatic analysis that suggest that these miRNAs have a somewhat redundant role in the same cellular processes that may result in a synergistic effect to promote CC progression. The results indicate that specific and common target genes for miR-23b-3p, miR-124-3p, and miR-218-5p regulate proliferation, migration, apoptosis, and angiogenesis, all processes that are related to CC maintenance and progression. Furthermore, several target genes may regulate cancer-related signaling pathways. We found that a total of 271 proteins encoded by the target mRNAs of miR-23b-3p, miR-124-3p, or miR-218-5p interact to regulate the cellular processes previously mentioned, and some of these proteins are regulated by HPV-16 E7. Taken together, information analysis indicates that miR-23b-3p, miR-124-3p, and miR-218-5p may potentiate their effects to modulate the cellular processes related to the progression and maintenance of CC with and without HPV-16 involvement.

## 1. Introduction

Cervical cancer (CC) is the fourth most common cancer diagnosed and the fourth cause of cancer death among women in the world. In 2020, 604,127 new cases of CC were diagnosed, and 341,831 deaths occurred due to this malignancy [1]. About 85% of deaths occur in developing or underdeveloped countries [2]. Most cases of CC are the result of persistent infection with genotypes of high-risk oncogenic human papillomavirus (HR-HPV), among which HPV-16 and HPV-18 are the most frequent [3,4,5]. The integration of viral DNA into the host cell genome and the overexpression of the E6 and E7 proteins of HR-HPV are key events for the initiation of cervical carcinogenesis. Viral oncoproteins, E6 and E7, directly or indirectly modulate the expression of multiple genes, which participate in the regulation of various stages of the viral cycle and cellular processes that contribute to cancer progression [2]. Through their interaction with various cellular proteins, E6 and E7 activate Fas, MAPK, Akt, PI3K, and Wnt signaling pathways, which modulate cellular proliferation, migration, invasion, and apoptosis, as well as epithelial–mesenchymal transition (EMT) and metastasis [6,7,8,9,10,11], cellular processes that modulate CC progression [12,13,14]. The dysregulation in the function of proteins, signaling pathways, and cellular processes, result from altered gene expression [15,16]. Viral oncoproteins, the accumulation of genetic and epigenetic alterations, and post-translational modifications lead to the inactivation of tumor suppressor genes, activation of oncogenes, and increase or decrease the expression of microRNAs (miRNAs) [17,18]. Alterations in miRNA expression are a causal factor in cancer progression and may be determinants of tumor aggressiveness [19]. 

MicroRNAs regulate the expression of several genes and thus modulate biological processes [20] such as cell proliferation, apoptosis, replicative immortality, immune response evasion, metabolic reprogramming, invasion, and metastasis [21,22,23,24,25]. On the other hand, messenger RNA (mRNA) contain different numbers of miRNA recognition elements (MREs), specific for individual miRNAs, in their 3′-UTR region. Thus, several miRNAs can hybridize with their specific MREs in one mRNA and a transcript can be simultaneously regulated by more than one miRNA [26]. In line with the above, miRNAs may play a synergistic or additive role in the cellular processes that they regulate [27,28,29].

In CC, the expression pattern of miRNAs is characterized by a decrease in tumor suppressor miRNAs and an increase in oncogenic miRNAs (oncomiRs) [30,31]. Previously, our research group analyzed the methylation status and expression of 22 miRNAs in cervical tissue from patients without squamous intraepithelial lesions (No-SIL) and negative for HPV infection, NonSIL HPV-16^+^, ^−^, low-grade premalignant lesions (LSIL), or HPV-16^+^ cervical cancer. The methylation status in the miR-124-2, miR-218-1, and miR-218-2 promoters increased as the severity of the lesions increased and was significantly higher in CC. The increase in methylation was related to a decrease in the relative expression of these miRNAs. No significant changes were found in the methylation of miR-23b [32]; however, miR-23b-3p was found to be decreased in CC tissues and cell lines [33]. Inhibition of methylation in the miR-23b-3p promoter induced increased miRNA expression and decreased proliferation, migration, and invasion of CaSki HPV-16^+^ cells. In C-33A HPV^-^ cells, increased expression of miR-23b-3p was associated with decreased proliferation and invasion. The results indicate that miR-23b-3p is a tumor suppressor that modulates CC progression [34]. On the other hand, Li et al. found low expression of miR-124-3p in biopsies and CC cell lines, and demonstrated that this miRNA regulates proliferation, invasion, and apoptosis [35]. Likewise, Liu et al. demonstrated that, in biopsies, HeLa HPV-18^+^ cells, C-33A, and SiHa HPV-16^+^ cells, miR-218-5p is under-expressed and regulates proliferation, migration, and invasion [36]. Negative regulation of miR-23b-3p, miR-124-3p, and miR-218-5p expression in CC tissues and cell lines with and without HPV suggests that the virus does not fully determine miRNA-tumor suppressive function.

This review focuses on the identification of cellular processes regulated by the target genes of miR-23b-3p, miR-124-3p, and miR-218-5p in cell lines and tissue from CC. Additionally, it provides information that documents the probability of synergistic or additive effects among specific target genes of each miRNA and targets common to two, or all three, miRNAs, which could enhance their effects on the regulation of biological processes and signaling pathways that promote the progression and maintenance of cancer.

## 2. miRNAs and Cervical Cancer Progression

Persistent HPV infections can result in intraepithelial lesions, classified histologically as grade 1 to 3 cervical intraepithelial neoplasia (CIN). Studies on the natural history of CIN3 reported that one in three CIN3 progresses to invasive cancer in 30 years [37]. 

Progression to invasion is poorly understood, but the processes involved are known to be complex and are promoted by various genetic and epigenetic alterations [17,38]. miRNAs are epigenetic regulators involved in each stage of evolution to invasive cervical cancer (ICC); however, little is known about the specific miRNAs involved in the deregulation of cellular processes that are altered during progression to ICC. Experimentally, it has been confirmed that some miRNAs regulate processes involved in the maintenance and progression of CC [30,31,39,40,41,42]. Some miRNAs are implicated in cervical carcinogenesis and in the progression of malignancy, by regulating targets that participate in proliferation, inhibition of apoptosis, EMT, migration, invasion, metastasis, and angiogenesis [40,43,44,45,46,47,48,49]. In the last five years, the study of miRNAs in CC has increased, many have been studied in cell lines and biopsies of patients with in situ cervical cancer (ISC) and/or ICC, with and without HPV infection. In vivo and in vitro studies confirm the participation of numerous tumor suppressor miRNAs and oncomiRs in the regulation of cell proliferation, inhibition of apoptosis, EMT, migration, invasion, and metastasis, as well as other cellular processes involved in cancer progression. In the literature published between 2015 and 2020, 42 original articles were found that report results on the expression of miRNAs and specific target genes, confirmed by luciferase assays. The investigations included biopsies from patients with CC, ICC, ISC, and HPV^+^ cell lines (SiHa HPV-16^+^, HeLa HPV-18^+^, and CaSki HPV-16^+^) and without HPV (C-33A). Tumor suppressor miRNAs were studied in 26 of these articles and in 5 of them (19.2%) miR-23b-3p, miR-124-3p, and miR-218-5p were the object of investigation (Table 1). In all five studies, the expression level of the three miRNAs was found to be decreased and consistent in tissues and cell lines, regardless of cancer stage (ISC or ICC), HPV infection (positive or negative), and viral genotype (HPV-16 or -18). Evidence indicates that, despite the influence of the factors mentioned, in CC, the level of miR-23b-3p, miR-124-3p, and miR-218-5p is lower than in normal tissue or in non-cancerous cell lines. Interestingly, through different target genes, the three miRNAs regulate proliferation, migration, invasion and apoptosis, all cellular processes that characterize CC progression (Table 1).

## 3. miR-23b-3p, miR-124-3p, and miR-218-5p Regulate Cervical Cancer Progression

miR-23b-3p, miR-124-3p, and miR-218-5p regulate different transcripts involved in processes related to the maintenance and progression of cancer [51]. The decrease of miR-23b-3p, miR-124-3p, and miR-218-5p in CC causes an increase in the level of their target mRNAs, including *MAPK1* and *c-Met*, *RGB2* and *AEG-1*, and *Gli3* and *ROBO1*, respectively, which promote cell proliferation, migration, and invasion, and inhibit apoptosis [35,44,52]. In CC cell lines C-33A, HeLa, SiHa, and CaSki, it was found that miR-23b-3p, miR-124-3p, and miR-218-5p are involved in the modulation of proliferation, EMT, migration, invasion, metastasis, and apoptosis, through the regulation of their target genes [20,32,34,46,53,54,55,56]. Li et al. found that, in HeLa cells, the overexpression of miR-23b-3p correlates with a decrease in cell proliferation and invasion and with an increase in apoptosis [44]. Likewise, the increase of miR-124-3p levels in HeLa and SiHa cells induces a decrease in cell proliferation and invasion, accompanied by an increase in apoptosis [35]. In a similar way, the upregulation of miR-218-5p decreases the proliferation, migration, and invasion processes of HeLa, SiHa, and C-33A cells [36]. These findings suggest that miR-23b-3p, miR-124-3p, and miR-218-5p are three tumor suppressor miRNAs that regulate the same cellular processes involved in CC progression.

In the research developed by our workgroup it was found that miR-23b-3p is decreased in HPV-16^+^ CC tissues and in C-33A, HeLa, and CaSki cells [32,33,34]. The ectopic overexpression of miR-23b-3p or the inhibition of methylation in the promoter region of miR-23b-3p significantly reduces *c-Met* expression in C-33A and CaSki cells. *c-Met* has five MREs for miR-23b-3p in its 3′-UTR region. c-Met is a tyrosine kinase receptor that activates Gab1 and FAK, and the signals stimulated by c-Met promote cell proliferation, migration, and invasion. A decrease in c-Met reduces proliferation, migration, and invasion in CaSki cells, as well as proliferation and invasion in C-33A cells [34]. On the other hand, in patients with CC, low levels of miR-23b-3p are associated with lower survival and predict a poor prognosis for women with this malignancy; restoration of miR-23b-3p expression reduces the activity of the AKT/mTOR signaling pathway and interferes with the progress of EMT, thereby decreasing the proliferation, migration, and invasion of SiHa and CaSki cells. *SIX1* is a direct target of miR-23b-3p and regulates, at least in part, the functional effects of this miRNA on CC progression [55]. Furthermore, the overexpression of miR-23b-3p induces a decrease in uPA protein levels, a urokinase that participates in the degradation of the extracellular matrix. In this way, this miRNA interferes with the migration of SiHa and CaSki cells [57].

The astrocyte elevated gene-1 (*AEG-1*), also known as metadherin (*MTDH*), is considered an oncogene whose overexpression contributes to carcinogenesis and tumor progression in various malignancies. In CC, *AEG-1* overexpression is associated with a poor prognosis. *AEG-1* is a direct target of miR-124-3p and has been found to be significantly increased in CC and in SiHa and HeLa cells; in contrast, miR-124-3p is decreased in CC tissue and in HeLa, SiHa, CaSki, and C-33A cells. In vitro, the restoration of miR-124-3p causes a decrease in *AEG-1*, along with decreased cellular proliferation, migration, and invasion, and a reduced progression of EMT in HeLa and SiHa cells. The increase in miR-124-3p correlates with an increase in E-cadherin and B-catenin and with low levels of N-cadherin, Twist, vimentin, and MMP9. Changes in the expression of these proteins indicate that miR-124-3p has a role in the regulation of EMT and metastasis. In line with this, the overexpression of miR-124-3p also results in a decrease in AKRIC2 and an increase in NF1, proteins involved in proliferation and migration in vitro and in vivo. Thus, changes in the expression of AEG-1/AKRIC2/NF1 may be part of the molecular mechanisms through which miR-124-3p reduces proliferation and migration [50,58]. High levels of miR-124-3p also reduce migration, invasion, and EMT in HeLa and C-33A cells through their interaction with AmotL1, an intercellular junction protein that regulates motility and promotes cell migration and invasion. *AmotL1* has been found to be overexpressed in CC [54]. Taken together, all the evidence indicates that miR-124-3p is deregulated in CC and that its target genes are directly involved in malignancy progression.

Data derived from various studies indicate that miR-218-5p also modulates several molecular mechanisms through which it contributes to CC progression. This miRNA is deregulated in CC cell lines and in tissue and, by restoring its expression in SiHa and HeLa cells, it reduces migration, invasion, and EMT through the negative regulation of *SFMBT1* and *DCUN1D1* [56]. miR-218-5p also regulates the expression of *LYN*, a tyrosine kinase that promotes cell growth and survival through the activation of NF-κB; when miR-218-5p decreases the level of *LYN*, migration, invasion, and EMT are reduced and apoptosis is increased in CC cells lines [59]. In a study performed on HeLa cells, the overexpression of miR-218-5p correlated with a decrease in TGF-β, VEGF, IL-6, PGE2, and COX-2, molecules that favor the immune escape of tumor cells. These results suggest that, in women with CC, the significant decrease in miR-218-5p promotes a state of immunosuppression and tumor cell survival. On the other hand, the transcript of the enzyme indoleamine 2,3-dioxygenase 1 (*IDO1*) is a direct target of miR-218-5p and, under conditions that dysregulate this miRNA, the expression of *IDO1* increases, promoting immunological tolerance to tumor cells. Additionally, miR-218-5p promotes apoptosis of HeLa cells by decreasing *survivin*, which negatively regulates caspase-3 [60]. Liang et al. analyzed the expression data of miRNAs obtained by high-throughput sequencing from 251 tissues with CC, registered in the cancer genome atlas (TCGA). They found that 78 miRNAs were differentially expressed (31 miRNAs were increased and 41 decreased) in CC tissues, with respect to normal tissues. In that study, low levels of miR-145 and miR-218-5p added to high expression of miR-200c predicted a poor prognosis for patients with CC and were significantly associated with average survival. By bioinformatic analysis, the researchers identified the target genes of miR-145, miR-218-5p, and miR-200c and, by using Gene Ontology (GO), found that the target mRNAs of these miRNAs regulate common biological processes. Functional enrichment analysis suggested that miR-145, miR-218-5p, and miR-200c target genes may be involved in several cancer-related pathways, including MAPK, AMPK, focal adhesion, cGMP-PKG, Wnt, and the mTOR signaling pathways [61]. 

A transcript can be regulated by two or more miRNAs and the evidence indicates that miR-23b-3p, miR-124-3p, and miR-218-5p are functionally related. Zare et al., by bioinformatic analysis, found that, in gastric cancer, miR-124-3p and miR-218-5p regulated the expression of *RUNX2*, a transcription factor whose function contributes to the modulation of cellular proliferation in this malignancy. Thus, *RUNX2* is a direct target of miR-23b-3p and miR-218-5p [62]. Results from independent studies, in ovarian cancer cell lines, also support the role of miR-23b-3p and miR-218-5p in *RUNX2* regulation. Ectopic expression of miR-23b-3p significantly inhibits cell proliferation, migration, and invasion by downregulating *RUNX2* [63]. Additionally, ectopic expression of miR-218-5p inhibits cell proliferation, colony formation, migration, and invasion in vitro and suppresses tumor growth in a tumor-bearing nude mouse model. Conversely, *RUNX2* overexpression rescues ovarian cancer cells from the suppressive effect of miR-218-5p, inducing proliferation, colony formation, migration, and invasion [64]. Thus, miR-23b-3p and miR-218-5p regulate the progression of ovarian cancer, in part, by repressing *RUNX2*. Other studies have also reported *RUNX2* as a direct target of miR-23b-3p [65], miR-124-3p [66], and miR-218-5p [67] in different types of cancer. The data described suggest that miR-23b-3p, miR-124-3p, and miR-218-5p converge in the regulation of cellular processes that contribute to the maintenance and progression of CC, through the regulation of specific targets of each miRNA and through target mRNAs shared by the three miRNAs. Thus, the function of miR-23b-3p, miR-124-3p, and miR-218-5p can have additive or synergistic effects on the modulation of gene expression and activation of signaling pathways, and, consequently, on the regulation of cellular processes recognized as cancer hallmarks. On one hand, it is known that one miRNA post-transcriptionally regulates more than 100 genes directly [68] and that this number increases by indirect regulation; on the other hand, an mRNA can be regulated by two or more miRNAs. Currently, there are few confirmed target mRNAs for miR-124-3p, miR-23b-3p, and miR-218-5p, and few proven target transcripts for two of these miRNAs, however, there are no studies that corroborate targets common to the three miRNAs in CC. Current bioinformatic tools can help explore these aspects.

## 4. miR-23b-3p, miR-124-3p, and miR-218-5p Target Prediction and Function Analysis

Experimental results and bioinformatic analysis with validation by luciferase assays indicate that two or more miRNAs simultaneously hybridize with the same transcript, due to the presence of more than one MRE in its 3′-UTR region. In this way, the miRNAs enhance their effect on cellular processes that regulate the maintenance and progression of different types of cancer, including CC [62,69,70,71].

**Figure 1:**
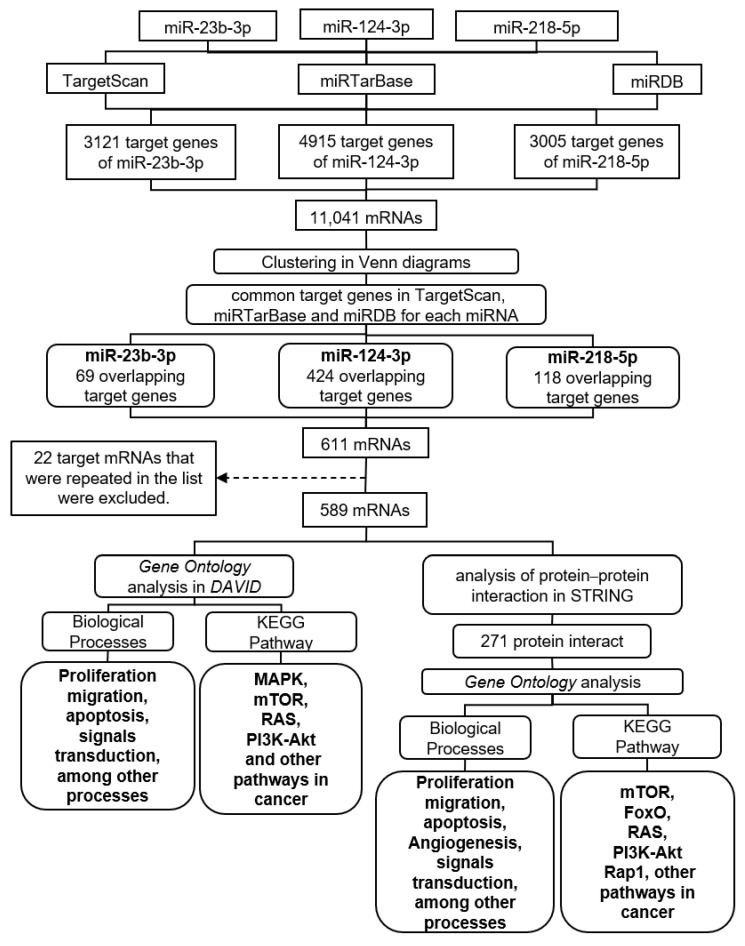
General flow diagram of the bioinformatics analysis for the identification of target genes of miR-23b-3p, miR-124-3p, and miR-218-5p, grouped and used for protein–protein interaction analysis and Gene Ontology.

miR-23b-3p, miR-124-3p, and miR-218-5p are tumor suppressors in CC and the data described suggest the probability that these miRNAs act additively or synergistically, enhancing their effect in the modulation of cellular processes that promote cancer progression. In order to explore the hypothesis that miR-23b-3p, miR-124-3p, and miR-218-5p have specific and shared targets involved in the regulation of the same cellular processes, and to generate information supporting the likelihood of synergistic or additive effects of the three miRNAs, a bioinformatic analysis strategy was designed (Figure 1).

The target genes of miR-23b-3p, miR-124-3p and miR-218-5p, were predicted using the TargetScan v.8.0 (http://www.targetscan.org/, accessed on 4 July 2022), miRDB v.6.0 (http://www.mirdb.org/miRDB/, accessed on 4 July 2022), and miRTarBase v.9.0 (http://mirtarbase.cuhk.edu.cn/php/index.php, accessed on 4 July 2022) tools. Table 2 shows that the number of target mRNAs for miR-23b-3p, miR-124-3p, or miR-218-5p varies between databases.

**Figure 2:**
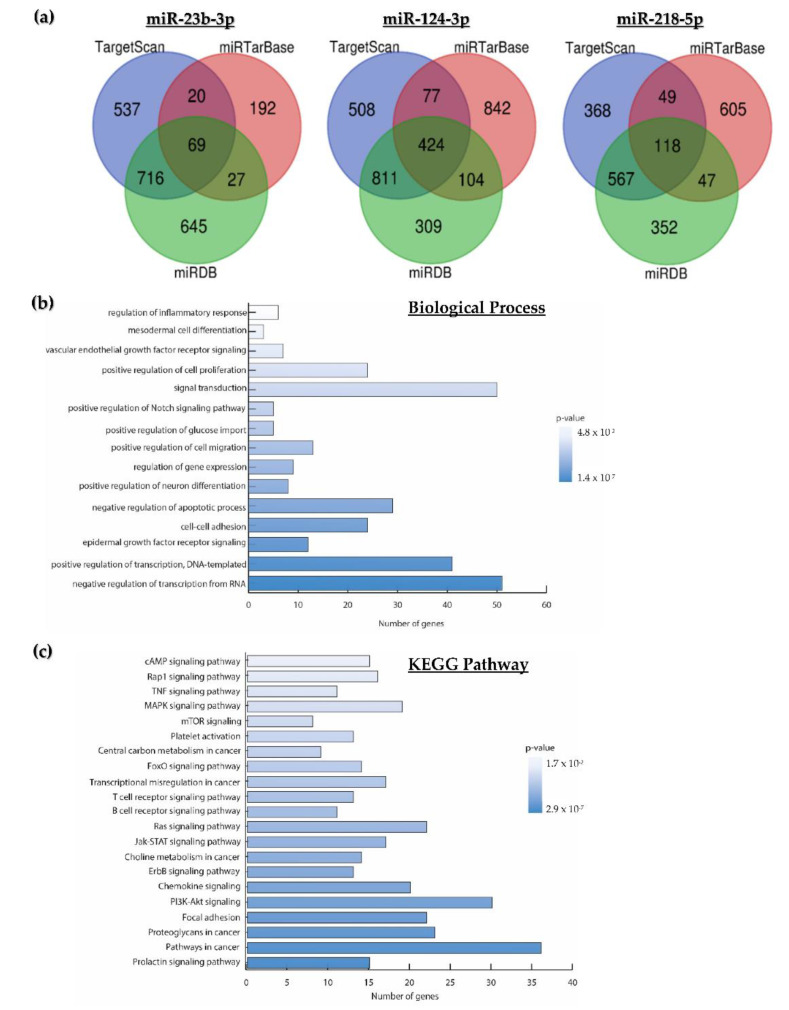
**Predicted target mRNAs for miR-23b-3p, miR-124-3p, or miR-218-5p and functional analysis. Overlapping transcripts were predicted using the TargetSan, miRTarBase, and miRDB tools.** (**a**) Predicted targets for miR-23b-3p, miR-124-3p, or miR-218-5p; (**b**) biological processes; and (**c**) signaling pathways significantly enriched by the target mRNAs of miR-23b-3p, miR-124-3p, and miR-218-5p.

The total targets for miR-23b-3p, miR-124-3p, and miR-218-5p found in the three databases were grouped in Venn diagrams. A total of 69 overlapping target mRNAs were identified for miR-23b-3p, 424 for miR-124-3p, and 118 for miR-218-5p (Figure 2a). Twenty-two of the 611 predicted transcripts were proposed by more than one program and were removed from the analysis. The remaining 589 were considered for Gene Ontology analysis on the DAVID 2021 platform (https://david.ncifcrf.gov/, accessed on 7 July 2022). The biological processes enriched by the target mRNAs of miR-23b-3p, miR-124-3p, and miR-218-5p were proliferation, migration, and apoptosis (Figure 2b). The enriched KEGG pathways included the MAPK, mTOR, RAS, and PI3K-Akt signaling pathways, among other pathways related to cancer (Figure 2c). 

**Figure 3:**
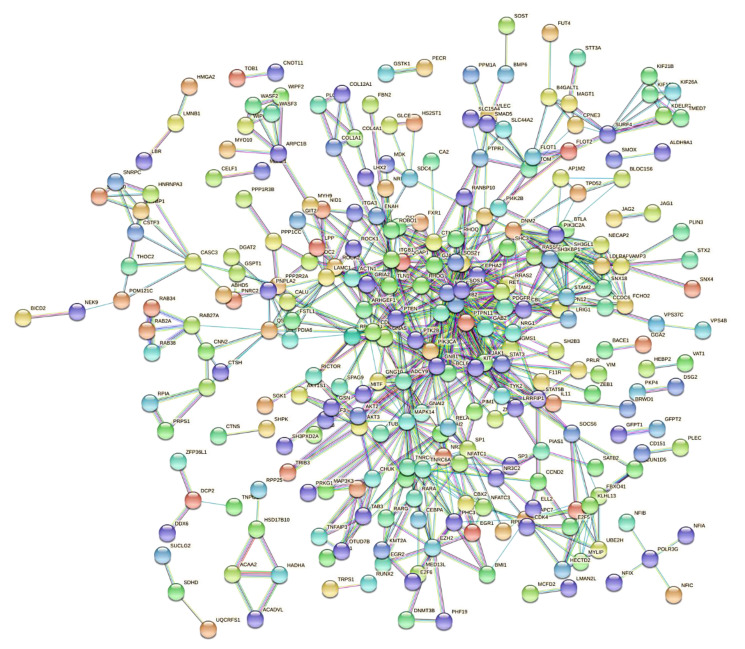
**Protein–protein interaction network.** The model shows that a total of 271 proteins encoded by the target mRNAs of miR-23b-3p, miR-124-3p, or miR-218-5p could interact to regulate cellular processes. The nodes represent the proteins, and the lines depict the interactions between them.

To verify whether the proteins encoded by the target transcripts of miR-23b-3p, miR-124-3p, and miR-218-5p are interrelated in the regulation of biological processes, protein–protein interaction networks (PPI) were built on the STRING platform (https://string-db.org/, accessed on 11 July 2022). From records in databases and experimentally determined interactions, the physical and functional interrelationships between the target proteins of the three miRNAs were investigated. For prediction, a confidence interval of 90% was defined, and it was found that of the 589 proteins included in the model, 271 interact with each other (Figure 3).

**Figure 4:**
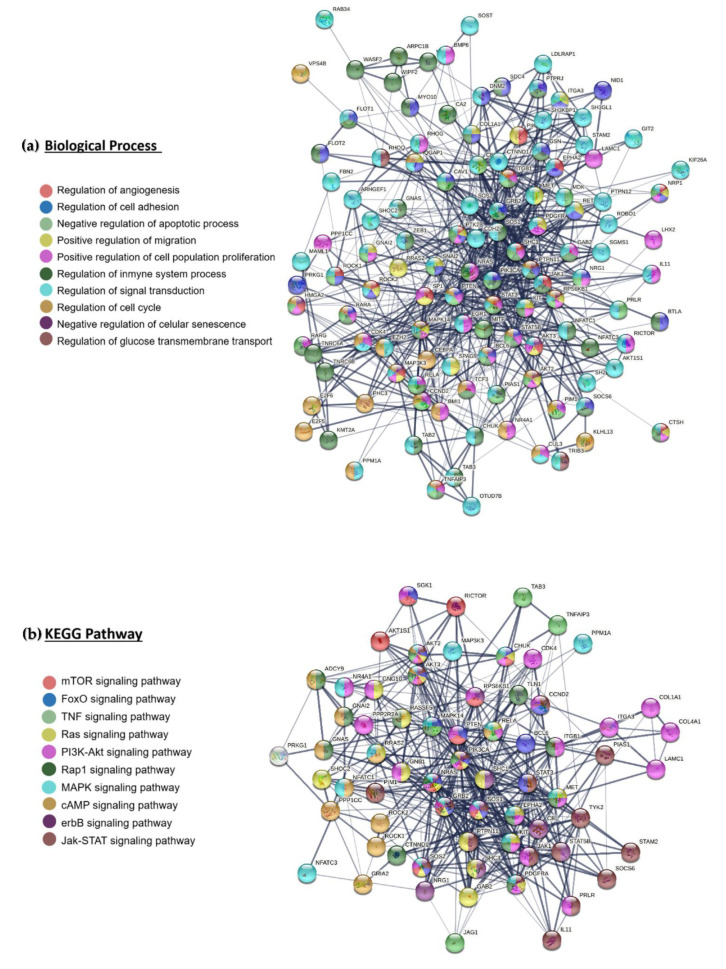
**Interactions between proteins regulated by miR-23b-3p, miR-124-3p, or miR-218-5p.** (**a**) 121 proteins participate in the regulation of biological processes and (**b**) 67 proteins interact to regulate signaling pathways.

Of the 271 proteins that interact, 121 participate in the regulation of cancer-related processes: angiogenesis, proliferation, migration, apoptosis, and the immune response. Some proteins participate in more than one process (Figure 4a). Sixty-seven of the 271 proteins participate in the MAPK, mTOR, and Ras signaling pathways, which are significantly enriched (Figure 4b).

**Figure 5:**
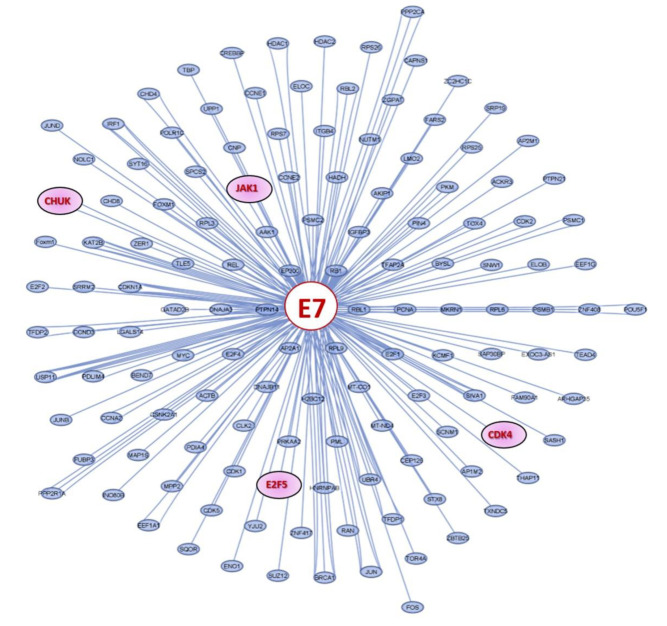
**Proteins regulated by HPV-16 E7.** The proteins encoded by the target mRNAs of miR-23b-3p, miR-124-3p, or miR-218-5p are colored in red.

The E6 and E7 proteins of HPV-16 alter the expression and activity of DNA-methyltransferase 1 (DNMT1), an enzyme that modifies cellular DNA methylation and, consequently, alters the expression of coding and non-coding genes, such as miRNAs [72,73,74]. Additionally, E6 and E7 of HPV-16 regulate the expression and function of various cellular proteins, which are targeted by tumor suppressor miRNAs [57,75]. To investigate whether some of the 271 proteins that interact in the PPI network are regulated by HPV-16 E6 or E7, the Cytoscape platform (https://cytoscape.org/, accessed on 12 July 2022) was used. The results indicate that E7 regulates CDK4, CHUK, E2F5, and JAK1, which participate in the modulation of cellular processes and signaling pathways related to cancer progression and maintenance (Figure 5).

**Figure 6:**
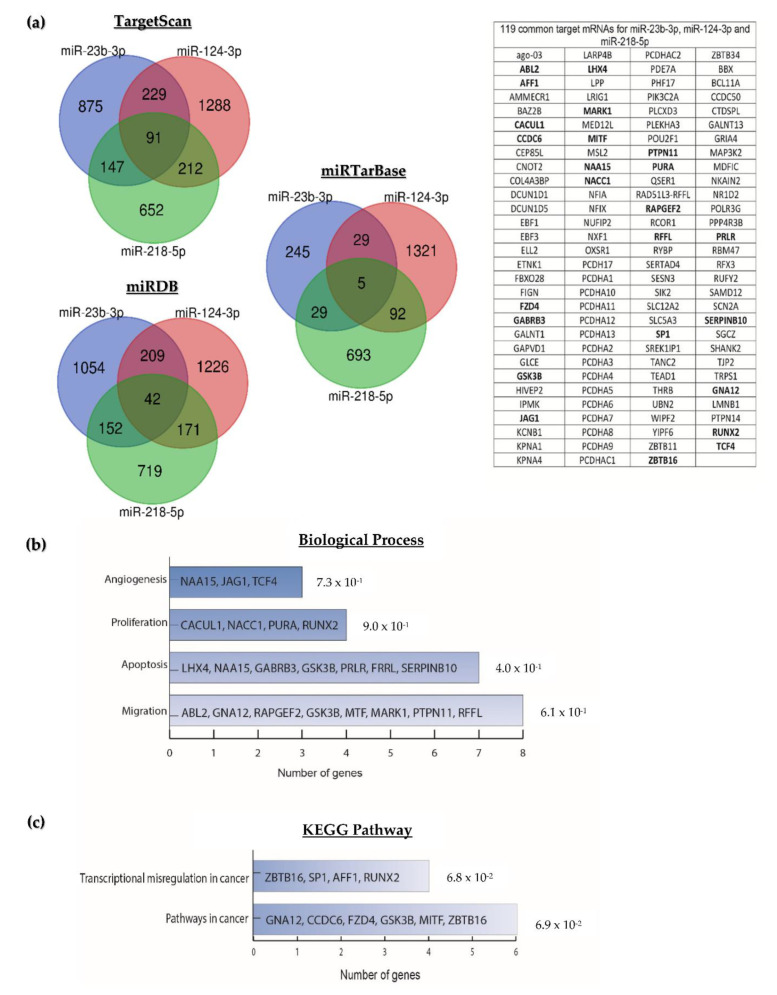
**miR-23b-3p, miR-124-3p, and miR-218-5p target mRNAs and enrichment analysis.** (**a**) Target transcripts shared between two or three miRNAs; (**b**) analysis of genetic ontology showing biological processes; and (**c**) signaling pathways in which the target mRNAs of miR-23b-3p, miR-124-3p, and miR-218-5p participate.

To explore whether miR-23b-3p, miR-124-3p, and miR-218-5p have shared target transcripts, the target mRNAs from each miRNA were grouped in Venn diagrams. TargetScan, miRDB, and miRTarBase predicted that 138 mRNAs are targets of the three miRNAs, 19 were redundant between programs and were removed from subsequent analyzes. One hundred nineteen shared target transcripts for miR-23b-3p, miR-124-3p, and miR-218-5p (Figure 6a) were subjected to enrichment analysis on the DAVID platform to investigate their biological function. No significant differences were found in the enrichment of the signaling pathways and the biological processes in which the target mRNAs of the three miRNAs participate; however, 24 transcripts participate in the regulation of proliferation, migration, apoptosis, and angiogenesis, all processes involved in the maintenance and progression of cancer (Figure 6b,c).

Some target mRNAs of miR-23b-3p, miR-124-3p, and miR-218-5p identified in this analysis, have been reported to be overexpressed in CC. The prolactin receptor (*PRLR*) is increased in CC cell lines and exerts an oncogenic function due to apoptosis inhibition [76]. The expression of *NACC1*, a transcriptional repressor that inhibits apoptosis and promotes cell proliferation, migration, and invasion, was found to be increased in CC tissue samples and cell lines [77]. *JAG1*, a ligand that activates the Notch signaling pathway, is overexpressed in HeLa and CaSki cells, where it promotes cell proliferation and migration and inhibits apoptosis [15]. Sun et al. reported that *SIK2*, a kinase protein, is increased in CC and that *SIK2* levels in SiHa and HeLa cells are related to an increase in the rate of proliferation, migration, and invasion and a decrease in apoptosis [78]. The transcription factor *SP1* is overexpressed in CC biopsies and increases cell proliferation and invasion in HeLa and CaSki cells [79].

*RUNX2* is a transcription factor that induces the expression of various genes involved in tumor progression, including *VEGF, MMP9, OPN*, *SNAI 1* and *2*, *TWIST1*, and *TIMP13* [80,81], and activates signaling pathways such as mTORC2, PTEN/PI3K/AKT, and NF-kB [67,81,82]. *RUNX2* is overexpressed and positively regulates the invasion of SiHa and C-33A cells [83]. *RUNX2* transcript is a direct target of miR-23b-3p [65], miR-124-3p [67], and miR-218-5p [65,66,67]. Using bioinformatic analysis (TargetScan v.8.0, http://www.targetscan.org, accessed on 20 July 2022) we confirmed that in the 3′-UTR region the *RUNX2* mRNA contains MREs with sequences complementary to the seed region of miR-23b-3p, miR-124-3p, and miR-218-5p (Table 3).

The information described is consistent with the hypothesis that miR-23b-3p, miR-124-3p, and miR-218-5p have additive effects or act in synergy in the regulation of processes that contribute to the progression of CC, and the data from bioinformatic analysis strengthen this proposal. GO analysis indicated that proliferation, migration, and apoptosis are the processes most enriched by targets of the three miRNAs. Of the 589 mRNAs that are targets of miR-23b-3p, miR-124-3p, and miR-218-5p, 119 are common targets of which 24 contribute to the regulation of proliferation, migration, apoptosis, and angiogenesis. Additionally, from the 271 proteins that interact in the PPI network, 121 are involved in angiogenesis, proliferation, migration, apoptosis, or immune response, and some proteins are involved in more than one process.

## 5. Conclusions

Despite the tremendous amount of research conducted on the initiation, maintenance, and progression of CC, tumor invasion and metastasis remain a colossal obstacle to treatment. Thus, studies on molecular mechanisms that regulate invasion and metastatic spread in CC are a priority. 

In CC tissues and cell lines, expression of miR-23b-3p, miR-124-3p, and miR-218-5p persists at low levels regardless of tumor stage, HPV infection, or viral genotype. It remains to be investigated whether these factors combine to deregulate the expression of these miRNAs. On the other hand, in CC cell lines it has been confirmed that, through specific targets, the three miRNAs regulate proliferation, migration, invasion, and apoptosis. In this regard, the question is, do miR-23b-3p, miR-124-3p, and miR-218-5p have synergistic or additive effects in the regulation of cellular processes and signaling pathways that promote cancer progression and maintenance?

Published information and results of bioinformatic analysis indicate that ectopic expression of miR-23b-3p, miR-124-3p, and miR-218-5p can lead to the rescue of altered cellular processes due to their tumor suppressor function and thereby reduce cell proliferation, migration, and invasion, inhibiting EMT and increasing apoptosis. The presence of specific MREs for each miRNA in the 3′-UTR region of the transcripts of two or more proteins that participate in the regulation of the same biological process or, simultaneously, in more than one function that contributes to cancer progression, invites us to hypothesize that miR-23b-3p, miR-124-3p, and miR-218-5p exert a functional synergistic or additive effect, through the negative regulation of their specific and shared target mRNAs. Additionally, the results of the bioinformatic analysis indicate that miR-23b-3p, miR-124-3p, and miR-218-5p have shared targets with key functions for CC progression. These data emphasize the need to experimentally verify whether mRNAs with MREs for all three miRNAs are simultaneously recognized by them and regulated in such a way as to potentiate the alteration in their biological expression and function. We suggest that miR-23b-3p, miR-124-3p, and miR-218-5p could potentiate their effects on the modulation of cellular processes related to the progression and maintenance of CC with and without HPV-16. The information described in this article summarizes the research generated around the expression and function of miR-23b-3p, miR-124-3p, and miR-218-5p and exposes the information gaps that still exist. The data generated from the bioinformatic analysis, and the experimental results described will guide future research on the gene regulation attributed to these miRNAs and will facilitate the identification of molecules with prognostic value and possible therapeutic targets. 

## Figures and Tables

**Table 1:** miRNAs involved in the progression of cervical cancer, detected in biopsies with or without HPV infection and in cell lines.

miRNA	Expression	Tissue/Cell Lines	Biopsies Available Information			Target mRNA	Biological Function	Reference
			Diagnostic	HPV Infection	Viral Genotype			
miR-23b-3p	Decreased	Biopsies (*n* = 16)/CaSki and C-33A	ISC	16 positive	16	*c-Met*	↑ Proliferation ↑ Migration ↑ Invasion	[34]
	Decreased	Biopsies/SiHa, HeLa, CaSki cells	ISC	NI	NI	*MAPK1*	↑ Proliferation ↑ Invasion ↓ Apoptosis	[44]
miR-124-3p	Decreased	Biopsies (*n* = 18)/HeLa, SiHa, CaSki and C-33A	11 ISC 7 ICC	10 positive 8 negative	NI	*AEG-1*	↑ Proliferation ↑ Migration ↑ Invasion ↓ Apoptosis	[50]
	Decreased	Biopsies/HeLa, SiHa, CaSki cells	ISC	22 positive	NI	*GRB2*	↑ Proliferation ↑ Invasion ↓ Apoptosis	[35]
miR-218-5p	Decreased	Biopsies (*n* = 80)/Hela, SiHa and C-33A	40 ISC 40 ICC	60 positive 20 negative	16/18	*ROBO1*	↑ Proliferation ↑ Migration ↑ Invasion	[36]

HPV = human papillomavirus; ICC = invasive cervical cancer; ISC = in situ cancer; *n* = sample size; NI = not informed by the authors; C-33A HPV^-^, SiHa HPV-16^+^, CaSki HPV-16^+^, and HeLa HPV-18^+^. ↑ biological function increased; ↓ biological function decreased. The miRNAs determined in patients with CC who had concomitant HIV infection were not considered.

**Table 2:** Target mRNAs with MREs specific for miR-23b-3p, miR-124-3p, and miR-218-5p.

Database	Number of Targets for Each miRNA		
	miR-23b-3p	miR-124-3p	miR-218-5p
TargetScanHuman	1342	1820	1102
miRTarBase	322	1447	819
miRDB	1457	1648	1084
Total	3121	4915	3005

**Table 3:** miRNA MREs specific for miR-23b-3p, miR-124-3p, and miR-218-5p.

	miRNA	Position in 3′-UTR Region	miRNA-mRNA Hybridization	Site
***RUNX2***	hsa-miR-23b-3p	1047–1068	miRNA 3′ CCAUUAGGGACCG-UUACACUA 5′ | | | | | | | mRNA 5′ AGUUCAUCCAGGCACAAUGUGAU 3′	7mer-m8
	hsa-miR-124-3p	1529–1535	miRNA 3′ CCGUAAGUGGCGCACGGAAU 5′ | | | | | | mRNA 5′ AGGGGAACCCCAAUCUGCCUUAC 3′	7mer-A1
	hsa-miR-218-5p	1308–1315	miRNA 3′ UGUACCAAUCUAGUUCGUGUU 5′ | | | | | | | mRNA 5′ UCAUAUUAAAAAGACAAGCACAA 3′	8mer
		2260–2266	miRNA 3′ UGUACCAAUCUAGUUCGUGUU 5′ | | | | | | | mRNA 5′ GUGUGUGGUAGCUUGAAGCACAC 3′	7mer-m8
		2840–2846	miRNA 3′ UGUACCAAUCUAGU—UCGUGUU 5′ | | | | | | mRNA 5′ CGUGGUUCUCUUUGUAGCACAA 3′	7mer-A1

Data obtained from TargetScan database.

## Data Availability

Not applicable.
